# On the potential of transauricular electrical stimulation to reduce visually induced motion sickness

**DOI:** 10.1038/s41598-023-29765-9

**Published:** 2023-02-25

**Authors:** Emmanuel Molefi, Ian McLoughlin, Ramaswamy Palaniappan

**Affiliations:** 1 School of Computing, University of Kent, Canterbury, UK; 2 ICT Cluster, Singapore Institute of Technology, Singapore, Singapore

**Keywords:** Rehabilitation, Electrocardiography - EKG, Nausea, Preclinical research, Translational research

## Abstract

Perturbations in the autonomic nervous system occur in individuals experiencing increasing levels of motion sickness. Here, we investigated the effects of transauricular electrical stimulation (tES) on autonomic function during visually induced motion sickness, through the analysis of spectral and time-frequency heart rate variability. To determine the efficacy of tES, we compared sham and tES conditions in a randomized, within-subjects, cross-over design in 14 healthy participants. We found that tES reduced motion sickness symptoms by significantly increasing normalized high-frequency (HF) power and decreasing both normalized low-frequency (LF) power and the power ratio of LF and HF components (LF/HF ratio). Furthermore, behavioral data recorded using the motion sickness assessment questionnaire (MSAQ) showed significant differences in decreased symptoms during tES compared to sham condition for the total MSAQ scores and, central and sopite categories of the MSAQ. Our preliminary findings suggest that by administering tES, parasympathetic modulation is increased, and autonomic imbalance induced by motion sickness is restored. This study provides first evidence that tES may have potential as a non-pharmacological neuromodulation tool to keep motion sickness at bay. Thus, these findings may have implications towards protecting people from becoming motion sick and possible accelerated recovery from the malady.

## Introduction

Motion sickness is an age-old physiological malady associated with epigastric discomfort, nausea and, in its severity, emesis. More than 2000 years ago, the Greek physician Hippocrates observed this physiological phenomenon^1^. Alas, motion sickness still poses as an aversive experience in modern transportation. Although being a technological advancement for humanity, the advent of autonomous and semiautonomous vehicles may well heighten the incidence and risk of motion sickness^2,3^. Moreover, digital devices and displays also pose an emerging hazard to many prone to the ailment^4^. 3D displays have also been found to evoke symptoms of motion sickness in contrast to their 2D counterparts^5^.

Explanation for the aetiology of motion sickness is not well understood. Hence many theories aiming to elucidate its mechanisms have been proposed^3^. Currently, a widely influential theory is one proposed by Reason and Brand^1^, defined as the sensory conflict theory. This theory posits that because of ambiguous sensory information from the eyes and the vestibular system, motion sickness is onset. Reason^6^ further developed the neural mismatch theory as a corollary to the sensory conflict theory. We now know from neuroimaging studies that this ambiguity in sensory information, particularly arising from visual stimulation, triggers prominent areas of the brain, for example the limbic system (known for regulating autonomic and endocrine function)^3,7^ and the insula (also involved in autonomic regulation)^8^. In addition, a motion video was shown to elicit autonomic changes leading to increased sympathetic and reduced parasympathetic activities of the autonomic nervous system (ANS)^9^. The symptomatology of motion sickness is characterised by a vast array of features which include, for instance, sweating, dizziness, drowsiness, headache, eyestrain, nausea and vomiting (a number of which are autonomically mediated).

Motion sickness changes ANS response; the ANS is a branch of the peripheral nervous system comprising the sympathetic and parasympathetic divisions. These physiological systems have been hypothesised to reflect low frequency power (LF) and high frequency power (HF) of the heart rate variability (HRV) signal; derived from electrocardiogram (ECG) measurements. Often the LF and HF metrics are expressed as a ratio (i.e., LF/HF), however, there is lack of agreement on the anatomical basis of the LF/HF ratio^10^. Reported findings on decreased HF power (HF; 0.15–0.40 Hz) of the HRV spectra in reponse to motion sickness have been consistent^11–13^. Furthermore, multiple studies have shown that the power ratio between low frequency (LF; 0.04–0.15 Hz) and HF band powers (LF/HF) increases with symptom development of motion sickness^12,14,15^. Given that the aforementioned metrics are features of altered physiological arousal by motion sickness, which has no cure, studying these changes in ANS response could enable researchers to manipulate them for non-pharmacologic interventions, in an effort to mitigate motion sickness.

Here we applied transauricular electrical stimulation (tES) non-invasively by sending electrical impulses from the tragus of the auricle to study its influence on motion sickness. Electrical stimulation of the auricular region has been found to have therapeutic effects in early research^16,17^. Additionally, by targeting this region there is a possibility of activating the vagus nerve^18^, which could serve as a conduit for balanced autonomic modulation. The vagus nerve (cranial nerve X) originates from the brainstem in an area called the medulla oblongata. From there it extends bilaterally receiving afferent signaling from the auricular branch, before travelling down the neck carrying parasympathetic innervation via the cervical branch, targeting major organs in the thorax, and making its way down to the gastrointestinal tract. It forms a complex neural network comprising afferent and efferent neural pathways. Conversely, it is possible that tES is not effectively able to activate the vagus nerve; hence, the current study does not address whether tES achieves its objective through vagus nerve stimulation or not. Recently, Sawada et al.^19^ showed that visually induced motion sickness from driving simulators can be reduced by, for example, coupling presentation of sound and vibration. Based on HRV analysis, Zhao et al.^20^ investigated the protective effects of transcutaneous electrical acustimulation (TEA) on motion sickness induced by a rotary chair. Here, we propose a non-invasive tES neuromodulation application to help in the management of motion sickness.

Our primary hypothesis is that by administering tES simultaneously with increasing levels of motion sickness, the metrics for LF and LF/HF ratio would decrease while the HF metric would increase. Furthermore, we hypothesized that the aforementioned objective measurements would be complemented by decreases in the scores of behavioral measurements obtained using an established and validated tool, the motion sickness assessment questionnaire (MSAQ). This is capable of measuring the severity of motion sickness in four dimensions (gastrointestinal, central, peripheral, sopite). Our rationale on the potential therapeutic/interventional effects of tES being that we may trigger a restoration in the autonomic imbalance observed during motion sickness.

## Results

We first sought to establish that the nauseogenic stimulation was inducing motion sickness by evaluating the HRV spectral parameters. Thus, baseline measurements were compared with “Nausea” measurements in the sham condition with the expectation that the parasympathetic tone would be decreased from baseline. Additionally, we expected the power ratio between LF and HF as measured by LF/HF ratio to increase from baseline.

**Figure 1:**
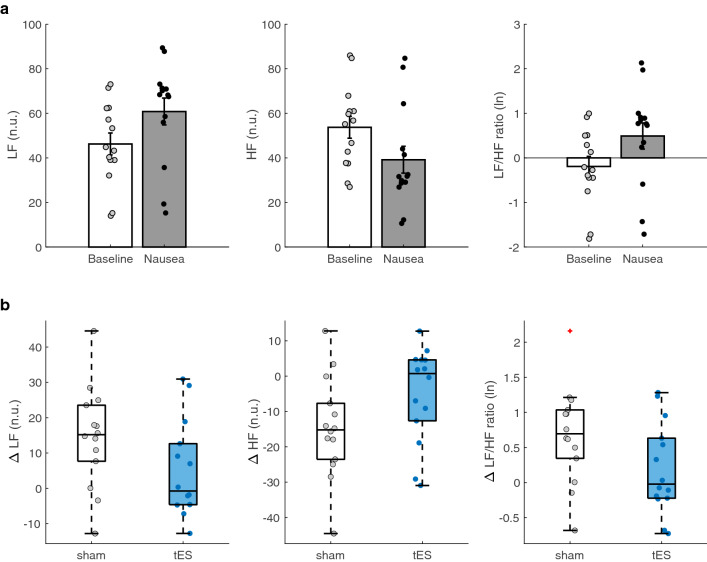
Spectral heart rate variability (HRV) measurements between participants at baseline and “Nausea” section, and between sham and transauricular electrical stimulation (tES) condition. **(a)** Average spectral power of low frequency (LF) and high frequency (HF), and LF/HF power ratio in response to nauseogenic stimuli. **(b)** Average change in LF, HF and LF/HF ratio in participants during sham and tES condition. Error bars represent standard error of the mean (SEM). The box plot central marks, box edges and whiskers represent medians, 25th to 75th percentiles and data range, respectively. The box plot also shows an outlier (red plus) in the sham condition for LF/HF ratio.

As expected, we did find a significant decrease in parasympathetic activity measured by HF n.u. (*t*$_{(13)}$ = 3.82, *p* = 0.0011) and increase in LF/HF ratio (*t*$_{(13)}= -3.71$ , *p* = 0.0013) as shown in (Fig. 1a). This is in line with previous studies that found decreases and increases in normalized HF and LF/HF ratio respectively with increasing symptoms of motion sickness. Further, a significant increase from baseline during nausea was found for LF n.u. (*t*$_{(13)}= -3.82$ , *p* = 0.0011). This result indicates that the nauseogenic stimulus was efficacious in eliciting robust physiological arousal.

The delta change of LF n.u. power and LF/HF ratio during nausea section and baseline was compared between sham and tES conditions (Fig. 1b). A significant decrease was found in LF n.u. (*t*$_{(13)}$ = 2.24, *p* = 0.0217) and LF/HF ratio (*t*$_{(13)}$ = 2.20, *p* = 0.0234) suggesting that tES was able to pull the level of autonomic arousal down. There was a significant increase in HF n.u. (*t*$_{(13)} = -2.24$, *p* = 0.0217) between sham and tES (Fig. 1b), indicating that tES was triggering an enhancement in parasympathetic modulation. The HF n.u. power for differences between the participants receiving sham before tES and those receiving tES before sham (i.e., *sham *$\rightarrow$ *tES* and *tES* $\rightarrow$ *sham*) did not appear to arise from fixed effect of order (*F*$_{(1)}$ = 0.54, *p* = 0.4782).

**Figure 2:**
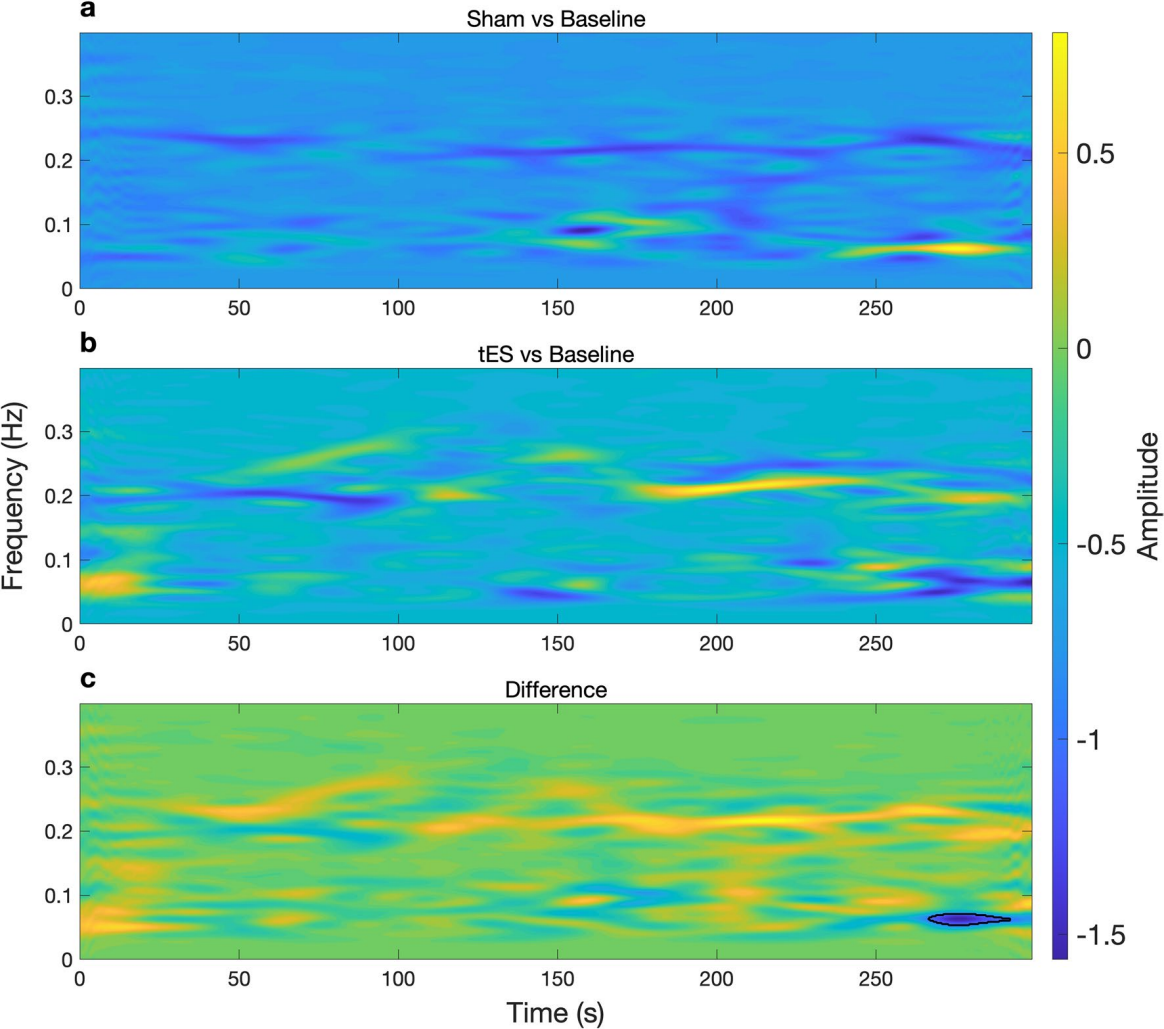
Time-varying power representations of heart rate variability (HRV) using the smoothed pseudo Wigner–Ville distribution (SPWVD) for an example participant. **(a)** Time-frequency power during sham (baseline subtracted) condition **(b)** and tES (baseline subtracted) condition. **(c)** Shows the time-frequency power difference between (Sham vs Baseline) and (tES vs Baseline). A statistical pixel-based permutation test using a pixel-level significance threshold of p $< 0.05$ revealed 1 cluster around 270–290 s (two-tailed non-parametric permutation tests). The significant cluster (region) is outlined by a black contour in **(c)**.

**Figure 3:**
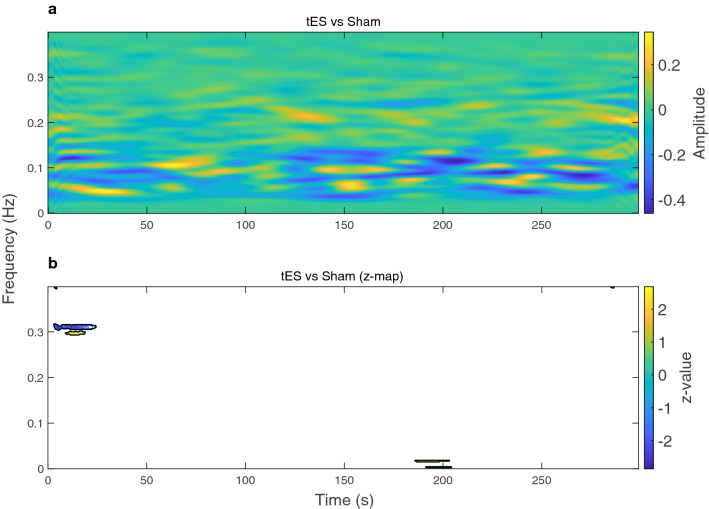
Time-varying power representations of heart rate variability (HRV) using the smoothed pseudo Wigner–Ville distribution (SPWVD) at the sample level. **(a)** Time-frequency power representation showing the power differences in tES and sham condition (after baseline subtraction within each condition). **(b)** Statistical z-map of the time-frequency power representation at the sample level, based on a cluster-level significance threshold p $< 0.05$ (two-tailed non-parametric permutation tests). Significant clusters (regions) are indicated by black contours on the statistical z-map.

The time-frequency representations of HRV were computed by performing smoothed pseudo Wigner-Ville distribution (SPWVD) for the sham and tES condition, and the difference between the two conditions (Figs. 2 and 3). SPWVD was used due to its ability to represent a signal in a robust manner in both time and frequency planes, circumventing trade-offs between time and frequency resolution. It also reveals dynamics of autonomic function during symptomatology development that may be associated with this complex syndrome. In Fig. 2, we show the time-frequency representation for an example participant. The blue colour in the time-frequency power map in Fig. 2a,b indicates that Baseline had higher power in those particular regions whereas it had lower power in the bright yellow regions. Note that (Sham vs Baseline) is interpreted as performing the operation (Sham–Baseline), similarly to other time-frequency maps shown in (Figs. 2 and 3). We observe that in Fig. 2a; Sham vs Baseline, the participant was showing attenuated HF power and increases in LF power for particular timepoints. While during (tES vs Baseline) the participant had a pronounced HF power at particular timepoints. The power difference between (tES vs Baseline) and (Sham vs Baseline) is shown in (Fig. 2c). After performing a pixel-based permutation test, a temporal cluster around 270–290 s was found to be statistically significant (*p* $< 0.05$). We performed cluster-based permutation tests at the sample level. The power differences at the sample level are shown in Fig. 3a; tES vs Sham. The resulting *z*-statistic map of (tES vs Sham) is shown in Fig. 3b with significant clusters outlined with a black contour. During tES stimulation, activity of HF HRV was increased (Fig. 3a); a cluster-based permutation test found two interpretable temporal clusters (cluster around 5–20 s, ${z} = -2.17$, *p* = 0.0150) and (cluster around 10–15 s, *z* = 2.12, *p* = 0.0170).

**Figure 4:**
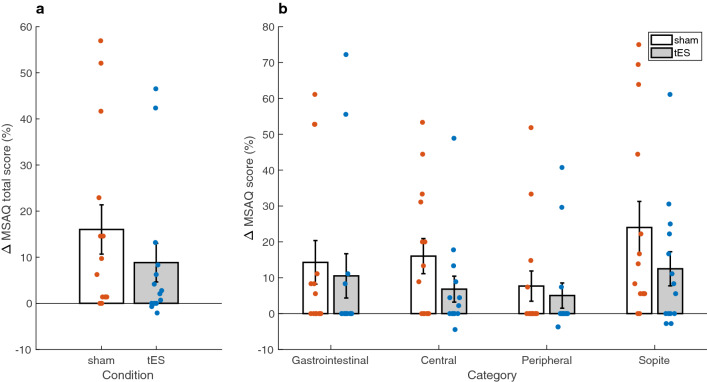
Summary of motion sickness assessment questionnaire (MSAQ) total and subscale scores across participants. **(a)** Average change in MSAQ total scores between sham and tES condition. **(b)** Average change in MSAQ scores within each category (gastrointestinal, central, peripheral, sopite) between sham and tES condition. Data are shown as mean ± SEM.

**Figure 5:**
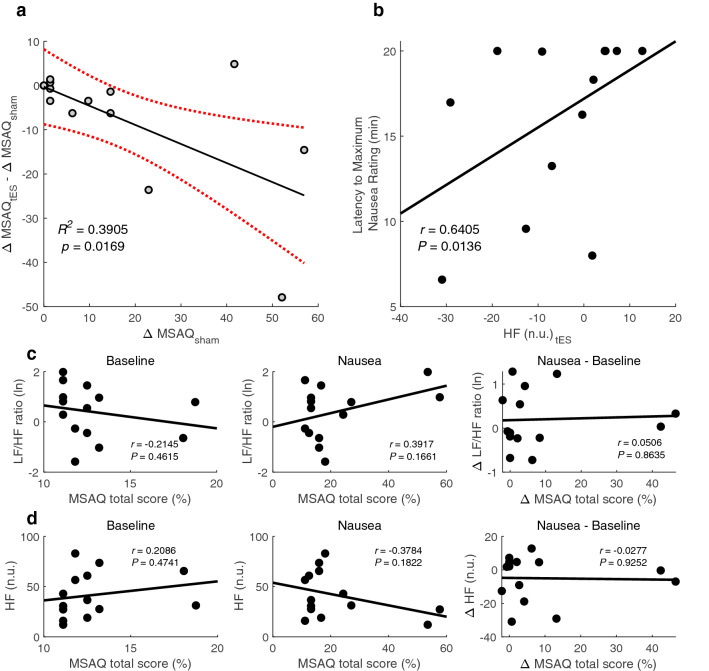
Scatterplots of linear regression, Spearman, and Pearson correlation analysis. **(a)** A linear regression between MSAQ during sham and the difference between tES and sham condition. **(b)** A Spearman correlation between HF power during tES condition and the latency to maximum nausea rating. **(c)** A Pearson correlation between the MSAQ total scores and log-transformed LF/HF ratio at Baseline and during tES as well as the difference between during and before tES intervention. **(d)** And MSAQ total scores and normalized HF at Baseline and during tES as well as the difference between during and before tES intervention. *r*, Spearman’s or Pearson’s correlation coefficient (*p*-values based on two-tailed statistical test).

The MSAQ scores were computed as delta change from baseline then compared between sham and tES conditions (Fig. 4). In addition to a Shapiro-Wilk normality test, histogram and normal probability visualisations were used as further tools to assess normality. Subsequently, statistical differences in the MSAQ data between sham and tES conditions were evaluated by a non-parametric Wilcoxon signed rank test. The MSAQ total score was found to be significantly lower in the tES condition compared to the sham condition (*p* = 0.0166) (Fig. 4a). A significant decrease was found in the Central (*p* = 0.0049) and Sopite (*p* = 0.0342) category subscores during tES compared to sham. The decrease in sickness symptoms that is observed in (Fig. 4b) for the Gastrointestinal and Peripheral category subscores did not reach statistical significance between sham and tES conditions (Gastrointestinal: *p* = 0.3125; Peripheral: *p* = 0.2188). Linear regression showed a link between MSAQ scores during sham condition and the difference between tES and sham condition ($R^2$ = 0.3905, *p* = 0.0169) (Fig. 5a). A positive relationship was observed between the latency to a maximum nausea rating and HF power during tES condition (Spearman’s rank correlation; *r* = 0.6405, *p* = 0.0136) (Fig. 5b). As presented in (Fig. 5c,d), we did not find any statistical differences in the relationships between LF/HF ratio and HF n.u. power after performing a Pearson’s correlation analyses. Although response bias and underreporting of malaise symptoms could be a plausible explanation for the lack of relationship, our small sample size may be an equally valid factor.

## Discussion

This pilot study provides initial insights into the therapeutic potential of tES for reducing visually induced motion sickness. We demonstrate that the spectra and time-varying features of HRV differ significantly when comparing sham and tES conditions. Whereby tES increases parasympathetic cardiac modulation and reduces the activity of the sympathetic nerves, supporting our hypothesis. Moreover, we show that the total MSAQ scores and MSAQ categorical subscores for the central and sopite dimensions were significantly lower in the tES condition when compared to the sham condition.

Because motion sickness has been implicated with decreased parasympathetic activity, and in turn contributes to perturbed autonomic function, we asked if tES could trigger a decrease in LF power while increasing HF power in order to reduce symptoms. While mechanistic underpinnings of electrical stimulation applied here are not fully parsed, evidence from studies done both in animals^21^ and humans^22,23^ indicate that the seesaw of autonomic function can be altered by electrical stimulation. The rationale therefore is that by targeting cutaneously accessible sensory (or somatic) receptors at the tragus of the auricle via electrical stimulation, we may alter the signal processing of the peripheral nerves to lower the stress on the nervous system caused by the build up of factors inducing motion sickness.

Parasympathetic neural activity was significantly greater in tES conditions compared to sham as measured by HF power (Fig. 1b). This suggests that participants were symptomatic in the sham condition and asymptomatic in the tES condition on the basis that decreased parasympathetic modulation has been found during increasing levels of motion sickness. Importantly, it indicates that tES enhanced relaxation in the physiology of the participants. Previous evidence showed that ANS response to nauseogenic stimuli changes autonomic function as if the nauseogenic stimulus is a threat; thereby triggering sympatho-excitatory circuits^9^. Moreover, Napadow et al.^7^ showed activation of the limbic system during presentation of a nauseogenic stimulus. We have long known that the threat and threat detection response is processed on components of the limbic system, which regulates e.g., autonomic function. Here, by introducing electrical sensory stimulation, sensory integration in the brain may be altered in a manner that suggests the nauseogenic stimuli seems innocuous. That is, the brain may be labeling sensory input from the nauseogenic stimuli as not ‘fearful’; therefore, leading to a balanced autonomic activity.

Previous work showed that electrical stimulation can reduce sympathetic nerve activity^23–25^, and therefore, yield parasympathetic predominance. These observations align with the present study, and here we show that individuals exposed to nauseogenic stimuli causing an aversive experience, have reduced sympathetic nerve outflow during tES intervention. This suggests tES may be eliciting effects of less agitation and as such, positively influencing autonomic response toward a desirable state of well-being. Parasympathetic and sympathetic systems can be considered foils of each other. Therefore, the explanation for parasympathetic activation from above, would mean inhibition of efferent sympathetic activity may be the reason we found reduced LF power, and consequentially, reduced LF/HF ratio (Fig. 1b).

In addition to HRV static spectral analysis, we computed the time-frequency features of HRV using SPWVD. Despite this being the first time, to the authors knowledge, that SPWVD is being applied in the context of motion sickness-associated ANS response; it may well allow us to discern the complexities of malaise progression. The time-varying power maps suggest motion sickness influences normal regulation of autonomic function by attenuating the activity of the HF component (Fig. 2a; Sham vs Baseline panel). Of note, this HF power attenuation appears to be portrayed by phasic alterations over time, perhaps simulating autonomic flushes that are implicated with cardiovagal bursts precursory nausea rating^14^. In Fig. 2b (tES vs Baseline panel), we observe the time-varying evolution of autonomic activity when tES is administered simultaneously with sickness-inducing stimuli. Interestingly, we observe that tES (Fig. 2c; difference panel) is able to fairly restore autonomic imbalance that we saw in the untreated (sham) condition (Fig. 2a; Sham vs Baseline panel). In particular, it is now easy to see the frequency component around 0.25 Hz that is driven by respiratory modulation. This respiratory modulation tends to be disrupted in the sham condition suggesting that motion sickness enhances lower frequency components while concurrently reducing parasympathetic modulation; indicating an increased level of autonomic arousal. Taken together, the time-frequency maps presented in (Figs. 2 and 3) may be useful to distinguish malaise severity between sham and tES.

Our findings from the MSAQ data showed significant decreases in the total scores and in all but two categorical subscores (gastrointestinal and peripheral) between sham and tES condition. Although there were no significant differences found for the gastrointestinal and peripheral category subscores, decreases were present as can be observed in (Fig. 4b). One possible explanation for this result could be an underreporting of sickness symptoms in the sham condition due to a negative emotion experienced from the nauseogenic stimulus. This could be driven by the markedly decreased MSAQ scores in the sopite category where we can find individual symptoms of ‘I felt annoyed/irritated’, ‘I felt drowsy’, ‘I felt tired/fatigued’ and ‘I felt uneasy’^26^. This dominance of sopite-like symptoms may have clouded participant judgement in reliably reporting how they felt after the experiment. Previous research has also suggested male participants may have a motion sickness response bias^27^. In addition, participants may have been cognitively constrained to reliably report their symptoms post-experiment as motion sickness is implicated with decreased cognitive performance^28^. Conceivably, the physiology of some participants may have been more responsive to the aversive experience of motion sickness and thus overshadowing the effects of tES. We have long known that susceptibility to the malaise of motion sickness is a highly individual experience^29^. Moreover, electrical stimulation has individual variability in and of itself^30^. The change in sham MSAQ total scores was a significant predictor of symptom outcome after tES intervention. That is, participants with more symptoms after sham, experienced a greater response to tES. This could be useful in individually targeted therapeutic intervention settings in order to help individuals with predispositions to the ailment. We also found a positive relationship between maximum nausea rating and HF power during tES. This suggests that during tES, participants tended to take longer to report a maximum nausea rating and thus finding it easier to cope with the nauseogenic visual stimulus.

To further understand tES potential to reduce motion sickness, we sought associations between autonomic and subjective changes using Pearson’s linear correlation coefficient. The lack of correlation between MSAQ total scores and observed HRV changes was somewhat surprising. While there is always a possibility that the relationship is complex and non-linear, we suggest caution when interpreting our findings. A more likely explanation is that the vagus nerve was not involved at all, and that the effects we found can be caused by any kind of electrical stimulation above sensory threshold. Future studies should take this into account.

Our study is not without limitations. First, the sample size in our study was very small, thus reducing the power for our statistical analysis. A second limitation is that sham sessions had no active stimulation; hence indicating that some participants could tell apart tES and sham conditions. We acknowledge that participants being aware of verum and sham stimulation could potentially bias the motion sickness-related changes in this study. Future studies should replicate these findings with different sham conditions that minimise potentially confounding effects of expectations. We note, however, that participants, were naïve to tES and sham stimulation electrode placement. A further limitation is that this study did not include a matching or sufficient number of male participants. The participant cohort was naïve to the nauseogenic stimuli and tES stimulation. Stimulation parameters utilised in this study were well-tolerated by all participants and no unexpected adverse effects were reported. While some participants prematurely stopped the presentation of nauseogenic stimulus due to a high severe feeling of nausea, no one vomited at the end of stimulus presentation. Future research is warranted where the findings here are augmented by the assessment of physiological correlates of motion sickness (i.e., neuroendocrine hormones such as arginine vasopressin and norepinephrine^31^). Moreover, the key questions to look forward to include; could we identify optimal stimulation parameters to improve effects of tES toward motion sickness reduction, and could increased stimulation duration improve the efficacy of tES to ameliorate motion sickness? In our future research, our investigations will extend to these questions and also administer an individually adjusted electrical current intensity.

In conclusion, insights from data in our pilot study showed that transauricular electrical stimulation may have potential on reducing the symptoms of visually induced motion sickness. With the caveats hitherto taken into consideration, we may be inching a step closer into preventing or slowing the onset of motion sickness, an incurable malady. Together, our findings suggest auricular electrical stimulation may hold promise for managing motion sickness, however we wish to emphasise that the autonomic data in this preliminary study were obtained from a very small number of participants. Future at-scale studies are necessary to confirm our results.

## Methods

### Participants

Sixteen healthy participants were recruited for the study. Of the 16 participants, 14 were retained (mean age ± S.D. = 26.7 ± 4.0 years, age range = 21–34 years, 12 females) for further analysis after two were excluded due to unsaved data and loss of follow-up respectively. Participant cohort had normal or corrected-to-normal vision. Inclusion criteria were no medical history of stroke, epilepsy or any neurological disorders. Additionally, participants were not using cardiac pacemakers, had no metal plates and were not on any medication. Participants received financial compensation ($\pounds 30$) in the form of an Amazon gift voucher for their participation. The methods were approved by the Central Research Ethics Advisory Group (ref: CREAG015-12-2021) of the University of Kent. Prior to participation, written informed consent was obtained from all participants. All study methods conformed to the principles set by the Declaration of Helsinki.

### Experimental protocol

**Figure 6:**
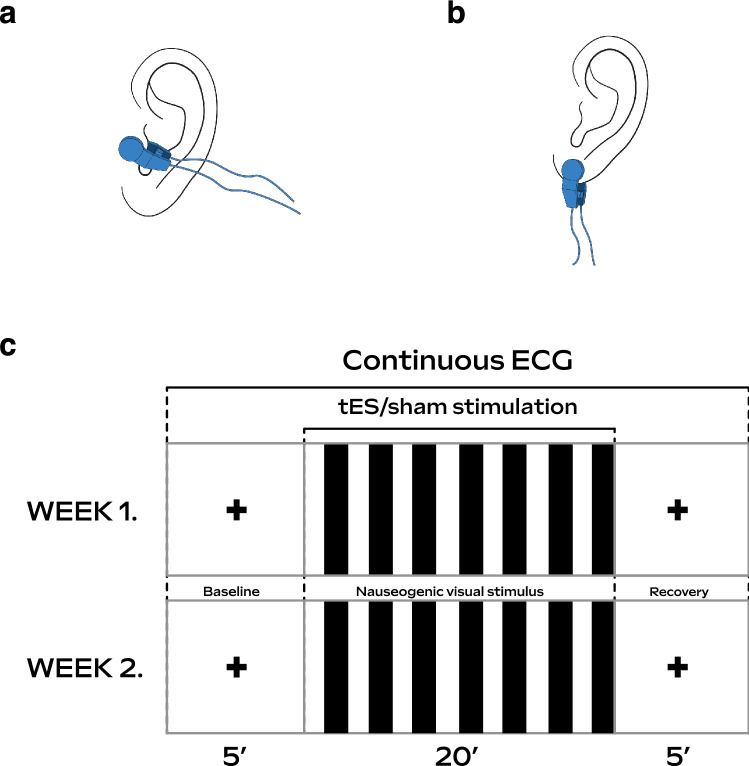
Experimental overview illustrations. **(a)** The electrode was clipped at the tragus site of the left ear during tES condition. **(b)** And clipped to the earlobe of the left ear during sham condition. **(c)** Participants underwent a baseline, nauseogenic visual stimulation and recovery section, respectively in both week 1 (first visit) and week 2 (follow-up visit), separated by 1 week. Electrocardiogram (ECG) signals were recorded continuously from beginning of baseline to end of recovery. Participants were randomly assigned to receive tES or sham in week 1 (first visit) and to receive opposite treatment on follow-up.

The experimental paradigm was designed as a within-participants cross-over study where each participant visited the lab for sham and active tES sessions. To allow for a washout period, sessions for sham and active tES were on separate days, with a minimum of 1 week in between. The order in which sham and active tES were administered was randomly assigned. Participants completed a pre- and post-MSAQ during both sessions. During both sham and tES sessions, participants observed a black crosshair projected at the centre of a screen for 5 min (baseline), followed by the Nauseogenic stimulus for a maximum of 20 min, and finally, a further black crosshair for 5 min (recovery) projected similar to baseline (Fig. 6c). Simultaneous to the presentation of the nauseogenic stimulus, the electrode of the tES stimulator was clipped to the earlobe of the left ear for sham sessions (Fig. 6b) and for tES sessions, to the tragus of the left ear (Fig. 6a). Participants provided subjective ratings to report their level of nausea by pressing on a keypad where (0 = “no nausea”), (1 = “mild”), (2 = “moderate”) and (3 = “strong”). We found that participants generally reached “mild” nausea at around 6 minutes relative to nauseogenic stimulus onset (Table 1). At the subjective level of experiencing a severe sensation of nausea, that is, when the participant had exceeded a rating of 3 (considered “strong”) and was on the verge of vomiting, the participant would stop the presentation of nauseogenic stimulus by pressing a button on a keypad. The computerised nauseogenic stimulus would then skip to the recovery section. The mean durations of the nauseogenic stimulus that participants received before pressing the button to quit the presentation were 13.2 (S.D. = 1.1 min, range = 12.4–14.4 min). Regardless of whether the participant stopped the stimulus prematurely or the 20 min elapsed, the recovery section always followed. From the beginning of the baseline section through to the end of the recovery section, ECG measurements were being recorded continuously. The researcher was with the participant in the lab during data acquisition proceedings, both to ensure the safety of the participant and to monitor smooth running of the experiment. Of note, the researcher was not in the view of the participant during the stimulus presentation. Participants were instructed to focus on the stimulus throughout, minimise conversation and to avoid body movements during the data acquisition process.

**Table 1:** Mean ± S.D. subjective nausea intensity ratings in minutes.

Subjective nausea ratings			
Condition	Mild nausea	Moderate nausea	Strong nausea
Sham	6.6 (4.6)	8.6 (5.9)	10.8 (6.1)
tES	6.3 (5.1)	10.5 (6.7)	12.3 (5.4)

The durations are given relative to stimulus onset.

### Nauseogenic stimulus

We created a nauseogenic visual stimulus to induce nausea. MATLAB (The MathWorks, Inc., Natick, MA, USA) software was used to develop source code for the nauseogenic visual stimulus using the Psychophysics Toolbox Version 3 (https://github.com/Psychtoolbox-3/Psychtoolbox-3)^32–34^. The stimulus was a horizontal translation of black and white vertical stripes with a circular shift of 62.5 °/s (Fig. 6c). This computerised stimulus simulates the visual input provided by the classic rotating optokinetic drum utilised to induce motion sickness/nausea^35–37^. It is well-known in the literature that this translation of alternating black and white stripes elicits illusory self-motion (vection) and nausea^38,39^. An fMRI compatible variant of the stimulus has been used effectively for inducing nausea in cardiac modulation and neuroimaging studies investigating motion sickness^7,8,13,14,40,41^. We presented the stimulus on a 47-inch LG LCD Widescreen (47LW450U, LG Electronics UK, UK) at a distance filling the participant’s full visual field. The display refresh rate was 60 Hz. The complete and contiguous study stimulus was a crosshair fixation section at baseline, nauseogenic stimulus section and a crosshair fixation section at recovery.

### Electrical stimulation

Non-invasive tES was applied using the EM6300A TENS (Med-Fit UK Ltd, Stockport, UK); a battery driven medical stimulation device with electrodes that can be clipped onto the ear (tragus or earlobe for example). Active tES was administered at the tragus site of the left ear (Fig. 6a); and sham (no actual stimulation) administered to the left earlobe (Fig. 6b). Stimulation current was delivered as asymmetric biphasic square-wave pulses with a pulse width of 200 µs, pulse frequency of 20 Hz and current intensity of 1.0 mA. Stimulation parameters were chosen based on literature assessing autonomic modulation. The parameters were tested prior to beginning each experimental session. The tES stimulation device was triggered manually by the researcher and had a countdown timer set for 20 min, in synchrony with the maximum duration of the nauseogenic stimulus. Moreover, the researcher turned off the device when the 20 min elapsed. In addition, the researcher immediately turned off the device for scenarios involving participants stopping the nauseogenic stimulus prematurely (see Experimental protocol for details on how premature stoppage of the nauseogenic stimulus was implemented). Perception of the above-mentioned stimulation parameters was reported by all participants without painful sensation. Our rationale for targeting the left ear was based on the fact that most previous studies had investigated with it^42^.

### ECG data acquisition, processing and analysis

Continuous ECG data acquisition was performed using a BioSemi ActiveTwo system (BioSemi B, V., Amsterdam, Netherlands). During the experiment, 64-channel electroencephalography (EEG) activity was also recorded. EEG data will be presented in a separate publication, allowing scope for discussion focused on ANS response for current analysis. The ECG signal was digitized at a sampling rate of 256 Hz using LabVIEW (National Instruments, USA) software. Electrode signal transduction was optimised by applying SIGNAGEL conductive gel. Electrode offset was within ± 10 µV. The recorded data was persisted as .bdf files for offline processing. ECG signal processing and analysis was performed using custom code developed in MATLAB software in accordance with recommended standards for HRV signals^43^. First, raw ECG data was visually inspected for any disturbances or distortions. Epochs of 5 minutes were extracted from the continuous ECG data for ‘Baseline’ and ‘Nausea’ sections. The logic for extracting epochs of the ‘Nausea’ section was as follows: if the participant completed the whole 20-min section without a subjective rating of at least 2 (moderate nausea), then the 5-minute epoch was obtained from right before the recovery section started. If the participant had a subjective rating of at least 2, then the 5-min epoch was obtained from right before the maximum rating was triggered. For scenarios where the participant prematurely stopped the nauseogenic stimulation due to a subjective severe feeling of nausea rating, then the 5-min epoch was obtained from right before the recovery section followed (note that the recovery section followed regardless of natural or subjective stoppage of the nauseogenic stimulation) - see Experimental protocol for technical implementation of nauseogenic stimulus premature stoppage. In essence, our data epoch selection was based on intertwining two methods that have been previously reported in the literature (i.e., percept- and condition-based analysis)^8,14,41^. Using the obtained 5-minute ECG epochs, we performed R-peak detection using the Pan-Tompkins algorithm^44^ and subsequently, the RR time-series (RR intervals) were generated. The *filtfilt* MATLAB function was used for implementation of ECG waveform filters in order to perform zero-phase digital filtering. Visual inspection was performed to ensure the quality of the RR series retained.

#### Frequency domain analysis

To perform spectral analysis of the HRV, we used the Lomb-Scargle periodogram to compute power spectral density (PSD) estimation on the obtained RR series. Subsequently, we computed the total power of HRV spectra (Total power; $\le$ 0.40 Hz), and the power of very low frequency (VLF; $\le$ 0.04 Hz), low frequency (LF; 0.04–0.15 Hz), high frequency (HF; 0.15–0.40 Hz) and the power ratio of LF to HF (LF/HF). Note that VLF power was only calculated to compute LF and HF in normalized units (n.u.) using formulae below^43^, and we took the natural logarithm (ln) of the LF/HF ratio.

1 $$\begin{aligned}{} & {} L{F}_{norm}=\frac{LF \; power}{Total \; power-VLF\; power}\times 100\ \end{aligned}$$

2 $$\begin{aligned}{} & {} H{F}_{norm}=\frac{HF \; power}{Total \; power-VLF\; power}\times 100\ \end{aligned}$$

3 $$\begin{aligned}{} & {} lnLF/H{F}_{ratio}={\rm{log}}\bigg (\frac{LF \;power}{HF\; power}\bigg ) \end{aligned}$$

#### Time-frequency representation analysis

We sought to perform time-frequency analysis knowing that this has the added advantage of observing how the frequency components of a signal varies over time. Thus, being able to observe the progression of symptoms as the participant suffers bouts of sickness may help elucidate how motion sickness evolves, in addition to how tES may be involved in the sickness evolution process. First, we applied a 4 Hz cubic spline interpolation for the resampling of the RR intervals into a uniformly sampled time series^14,45^. The *interp1* MATLAB function was used for implementation of cubic spline interpolation with the interpolation method set to “spline”. Resampling in HRV literature is mostly performed before frequency/time-frequency decomposition when methods such as those based on Fourier transforms, or wavelet-based PSD estimates are applied (i.e., these approaches assume uniform sampling)^41,43^. It should be noted that other resampling methods such as linear interpolation are possible. The Hilbert transform was applied to obtain the analytic signal association to the HRV signal. Then we performed smoothed pseudo Wigner-Ville distribution (SPWVD) to compute time-frequency power. The SPWVD is a well-known method for its good time-frequency resolution and robustness towards cardiovascular signal analysis^46^. Previous studies have utilised the SPWVD method to detect obstructive sleep apnea (OSA) in ECG recordings (e.g.,^47^) in addition to drowsiness detection (e.g.,^45^). The SPWVD is a member of the Cohen’s class family of time-frequency distributions^48^, and can be defined as follows:

4 $$\begin{aligned} SPWVD_{x}^{g,H} \left( {t,f}\right) =\int _{-\infty }^{\infty }g\left( {t}\right) H\left( {f}\right) x\left( {t+\frac{\tau }{2}}\right) x^{*}\left( {t-\frac{\tau }{2}}\right) e^{-j2\pi ft}d\tau \end{aligned}$$

The term *g*(*t*) represents performing a convolution (smoothing) in time. Whereas, the term *H*(*f*) represents spectral smoothing of *g*(*t*) using a nonparametric fast Fourier transformation (FFT). Of utmost importance, by looking at the local features of the signal *x* in time *t*, SPWVD enables independent smoothing in time and in frequency^49^.

### Motion sickness questionnaire

In order to assess symptoms of motion sickness, participants completed the motion sickness assessment questionnaire (MSAQ)^26^ before and after the experiment. The MSAQ is a well-known validated tool consisting of 16 symptoms categorised into 4 dimensions of motion sickness defined as gastrointestinal, central, peripheral and sopite-related. The individual symptoms are rated on a nine-point scale where (1 = “not at all”) and (9 = “severely”). The MSAQ total score is computed as a percentage of summed points from all symptoms. While scores for the four categories are percentages of summed points from within each category.

### Statistical analysis

All statistical analyses were performed using MATLAB software. We reported variables of the HRV spectra and the MSAQ as means with standard error (SEM). Changes of HRV spectral data were analysed using a paired one-tailed *t*-test to assess the effects of (1) “Baseline” and “Nausea” measurements in the sham condition, and (2) comparisons between sham and tES condition. We computed Pearson’s correlation coefficient between the MSAQ subjective total scores and the log-transformed LF/HF ratio and normalized HF power (two-tailed). Relationship between the latency to a maximum nausea rating and HF power was tested using Spearman’s rank correlation coefficient (two-tailed). Linear regression was performed on MSAQ data using the *fitlm* MATLAB function. Time-frequency representations (TFR) of SPWVD were examined using non-parametric permutation testing to analyse differences between sham and tES condition. We note that the idea of permutation-based statistics is heavily used in neuroscience, however, the methods are amenable to statistical analysis of time-frequency matrices. Early, neuroscience focused research comprehensively detailed the theory and justifications of permutation statistics^50,51^. All our permutation-based statistical tests were two-tailed. At the participant level, we examined TFR power using pixel-based permutation statistics (i.e., to correct for multiple comparisons). At each of the 1000 random permutations, we extracted the two most extreme values (i.e., minimum and maximum) from the TFR difference matrix to generate a null distribution. Then our threshold was determined by taking the 2.5th and 97.5th percentiles from our distribution, applying *p* = 0.05. Clusters found using this statistical method can be observed in (Fig. 2c). We used non-parametric cluster-based permutation tests^52^ to examine the differences in the time-frequency representations of SPWVD at the sample level between sham and tES condition. Herein, it is important to note the necessity of cluster-based permutation tests on time-frequency data. From the basis of a significance threshold at (*p* = 0.05), we computed a z-value, which, in turn, was used to threshold the z-transformed time-frequency power difference between tES and sham condition. To generate a null distribution, we performed 1000 random permutations, where at each permutation extracting the maximum cluster mean of the z-values. Our analysis is based on discussing the temporal clusters found as shown in (Fig. 3b). However, it is worthy of caution that clusters identified using this method may represent effects detected though maybe not supported by the permutation test^53^. A difference worthy of note between cluster and extreme-pixel correction is that, cluster correction tends to support bigger clusters. Whereas, pixel-based correction is more stringent though it can detect smaller clusters or single pixels. Hence, our rationale for using cluster correction at the sample level. The changes in the MSAQ were assessed for normality using the Shapiro–Wilk test^54^ and subsequently analysed using a one-tailed non-parametric Wilcoxon signed rank test. Statistical significance was considered at (*p*< 0.05).

## Data Availability

The datasets generated and analysed during the current study and the analysis code are available from the corresponding author upon reasonable request.
